# Prospective Study of a Multimodal Convulsive Seizure Detection Wearable System on Pediatric and Adult Patients in the Epilepsy Monitoring Unit

**DOI:** 10.3389/fneur.2021.724904

**Published:** 2021-08-18

**Authors:** Francesco Onorati, Giulia Regalia, Chiara Caborni, W. Curt LaFrance, Andrew S. Blum, Jonathan Bidwell, Paola De Liso, Rima El Atrache, Tobias Loddenkemper, Fatemeh Mohammadpour-Touserkani, Rani A. Sarkis, Daniel Friedman, Jay Jeschke, Rosalind Picard

**Affiliations:** ^1^Empatica, Inc., Boston, MA, United States; ^2^Division of Neuropsychiatry and Behavioral Neurology, Rhode Island Hospital, Brown University, Providence, RI, United States; ^3^Department of Neurology, Rhode Island Hospital, Brown University, Providence, RI, United States; ^4^Harvard Medical School, Boston, MA, United States; ^5^Department of Neuroscience, Bambino Gesù Children's Hospital, Istituto di Ricovero e Cura a Carattere Scientifico, Rome, Italy; ^6^Department of Neurology, Boston Children's Hospital, Boston, MA, United States; ^7^Department of Neurology, Downstate Medical Center, State University of New York, Brooklyn, NY, United States; ^8^Department of Neurology, Brigham and Women's Hospital, Boston, MA, United States; ^9^Department of Neurology, New York University Langone Medical Center, New York, NY, United States; ^10^MIT Media Lab, Massachusetts Institute of Technology, Cambridge, MA, United States

**Keywords:** epilepsy, seizure detection, wearable sensors, machine learning, clinical validation

## Abstract

**Background:** Using machine learning to combine wrist accelerometer (ACM) and electrodermal activity (EDA) has been shown effective to detect primarily and secondarily generalized tonic-clonic seizures, here termed as convulsive seizures (CS). A prospective study was conducted for the FDA clearance of an ACM and EDA-based CS-detection device based on a predefined machine learning algorithm. Here we present its performance on pediatric and adult patients in epilepsy monitoring units (EMUs).

**Methods:** Patients diagnosed with epilepsy participated in a prospective multi-center clinical study. Three board-certified neurologists independently labeled CS from video-EEG. The Detection Algorithm was evaluated in terms of Sensitivity and false alarm rate per 24 h-worn (FAR) on all the data and on only periods of rest. Performance were analyzed also applying the Detection Algorithm offline, with a less sensitive but more specific parameters configuration (“Active mode”).

**Results:** Data from 152 patients (429 days) were used for performance evaluation (85 pediatric aged 6–20 years, and 67 adult aged 21–63 years). Thirty-six patients (18 pediatric) experienced a total of 66 CS (35 pediatric). The Sensitivity (corrected for clustered data) was 0.92, with a 95% confidence interval (CI) of [0.85-1.00] for the pediatric population, not significantly different (*p* > 0.05) from the adult population's Sensitivity (0.94, CI: [0.89–1.00]). The FAR on the pediatric population was 1.26 (CI: [0.87–1.73]), higher (*p* < 0.001) than in the adult population (0.57, CI: [0.36–0.81]). Using the Active mode, the FAR decreased by 68% while reducing Sensitivity to 0.95 across the population. During rest periods, the FAR's were 0 for all patients, lower than during activity periods (*p* < 0.001).

**Conclusions:** Performance complies with FDA's requirements of a lower bound of CI for Sensitivity higher than 0.7 and of a FAR lower than 2, for both age groups. The pediatric FAR was higher than the adult FAR, likely due to higher pediatric activity. The high Sensitivity and precision (having no false alarms) during sleep might help mitigate SUDEP risk by summoning caregiver intervention. The Active mode may be advantageous for some patients, reducing the impact of the FAR on daily life. Future work will examine the performance and usability outside of EMUs.

## Introduction

Generalized tonic-clonic seizures and focal-to-bilateral tonic-clonic seizures are the most dangerous types of seizures and represent major risk factors for sudden unexpected death in epilepsy (SUDEP), especially when patients are left unattended, e.g., nighttime (1–4). Beyond the risk of serious or life-threatening injuries (5), the lives of patients and their caregivers are heavily influenced by the unpredictability of seizures, which results in decreased quality of life and contributes to social isolation, especially in adolescents (6, 7).

Over the past decade, wearable devices equipped with automated seizure detection algorithms have been suggested to complement and overcome limitations of the gold standard video-electroencephalography (v-EEG) performed in the Epilepsy Monitoring Unit (EMU) (8–12). Such devices target a continuous, remote, unobtrusive and less expensive monitoring of patients. They are useful mainly for two reasons: (i) to prompt caregivers' intervention during or shortly after a seizure while the patient is unattended, when the risk of injury and SUDEP is the highest (13), consequently relieving both patients and caregivers; (ii) to provide objective and more accurate seizure counts in outpatient settings, overcoming the limitations of seizure diaries (14, 15). Several surveys have demonstrated the need for accurate wearable seizure detection (16–22).

Most of the proposed systems can detect seizures with a clear motor activity component; however, they can have high false alarm rates (FAR) (11, 23). Among the non-EEG seizure-monitoring devices, multimodal systems hold the most promise for attaining both high sensitivity and a low number of false alerts (24, 25). Moreover, these systems have the potential to assess seizure severity by tracking and analyzing multiple bio-signals in the peri-ictal period (13, 26, 27).

Growing efforts have been made by the scientific and clinical community to standardize the studies on wearable seizure detection devices to perform a rigorous validation and to enable their ubiquitous adoption in outpatient settings (24, 28, 29). Published guidelines have tried to adapt the STARD criteria to the specific use case of seizure detection (30). The main recommendation is to test the performance of seizure detection devices during prospective “phase III” multi-center EMU studies and “phase IV” in-field studies, where the detection algorithm is “fixed-and-frozen” on a set of patients' data previously recorded from a dataset completely different from the Test Cohort (28, 29). A few studies have been published that fulfilled phase III or IV criteria, using a dedicated device and fixed algorithm (31–35). Only one of them used multimodal seizure detection (32) and it was tested only during nighttime and only on a group of patients that included some of the same people used to develop the algorithm, two conditions that can inflate the algorithm's performance. There is a need for studies examining the 24-h performance of multimodal devices on independent data sets, which do not include any patients used when developing the algorithm.

In this work, we present an evaluation using a prospective, multi-center study with 24-h data from an independent group of patients wearing multimodal wrist-worn devices combining accelerometers (ACM) and electrodermal activity (EDA) sensors. We evaluate the detection of two seizure types, i.e., “focal onset to bilateral/unilateral tonic-clonic” (FBTC) seizures, previously known as secondary generalized tonic-clonic seizure, and “generalized onset tonic-clonic” (GTC) seizures, previously defined as primary generalized tonic-clonic seizures. For brevity, we will use “convulsive seizures” (CS) to generically refer to the two seizure types included in the study. The ACM with EDA sensor combination has been shown as promising to capture signs of ongoing CS (36–38), leading to the commercialization of a wristband specifically designed to provide real-time alerts of detected CS (Embrace wristband, Empatica Inc). A previous multicenter study reported high sensitivity (52 of 55 CS detected) and low false alarm rate (1 false alarm every 5 days) using a machine learning algorithm (38) that outperformed the pioneering state-of-the-art ACM and EDA system (37) in a direct performance comparison, both using independent 24-h test data. However, the study qualified for phase II, as it reported a cross-validation analysis, meaning that the parameters of the algorithm were not the same for all the analyzed patients (29). Here we report the performance of a “fixed-and-frozen” algorithm on a Test Cohort of participants that are non-overlapping with participants used in the training dataset. Three main sets of analyses are shown in this study:

1. Performance in detecting CS on the whole (24-h a day, all ages) dataset and on pediatric and adult populations, separately, which provides indications on potential implications of age on the CS detection effectiveness.

2. Performance in detecting CS during low-motion conditions, i.e., on periods of sedentary behavior, which tend to be sleep periods associated with greater isolation and SUDEP risk.

3. Performance in detecting CS with two operating points, i.e., the FDA-cleared^1^ settings and a less sensitive Active operating point, to provide indications on the potential of the algorithm to be adapted according to different populations or individual needs and expectations.

## Materials and Methods

### Study Design and Endpoints

This is a prospective, non-randomized multi-site EMU clinical trial undertaken to get the clearance by the US Food and Drug Administration (FDA) of an investigational monitoring and alerting system for the identification of specific types of seizures (i.e., CS) using a device worn on the wrist. The device embeds a Detection Algorithm that processes 3-axis ACM and EDA sensor data to detect CS events. As per the requirements by the FDA on medical software, the Detection Algorithm must be “fixed-and-frozen.”

The population intended for the usage of the device included children age 6 (included) to 20 (included) and adults age 21 (included) and up.

The performance of the fixed-and-frozen Detection Algorithm on the Test Cohort has been evaluated in terms of sensitivity (or percent positive agreement) as the primary endpoint, and false alarm rate per 24 h-worn (FAR) as the secondary endpoint. The primary endpoint for the clinical validation of the wearable medical device was to reach a lower bound of the 95% confidence interval of the sensitivity higher than 0.7, for pediatric and adult groups, separately. The secondary endpoint required that the Detection Algorithm reached a FAR lower than 2 false alarms per day, for pediatric and adult groups, separately.

### Data Development Plan

#### Clinical Sites

The clinical sites involved in the study are members of the National Association of Epilepsy Centers (NAEC) certified as level IV in the USA, or members of LICE (Italian League against Epilepsy) and advanced epilepsy center^2^ in Italy. From 2014 to 2018, a series of IRB approved research studies using ACM and EDA wrist-worn devices were carried on in several Level IV NAEC members in the US, including Boston Children's Hospital (CHB), New York Langone Medical Center (NYU), Emory Healthcare (EMORY), Children's Hospital of Atlanta (CHOA), and Rhode Island Hospital (RIH). The studies were conducted following US government research regulations, and applicable international standards of Good Clinical Practice, and institutional research policies and procedures. Additionally, from 2017 to August 2018, a Pivotal study was conducted in a Level IV center in the US, namely New York Langone Medical Center (NYUP^3^) and in an advanced epilepsy center in Rome, Italy, Ospedale Pediatrico Bambin Gesú (OPBGP)^4^. The collected labeled data have been used to test the Detection Algorithm. The timeline of data collection at each clinical center is reported in the Supplementary Figure 1.

#### Sample Size Estimation

The estimation of a minimum required sample size was based on the Sensitivity requirements of the primary endpoint, i.e., meaning that we computed the minimum required number of patients experiencing at least one convulsive seizure during admission. The sample size was computed taking into account the possible presence of multiple events for the same subject (clustered data) and the need for a high value of sensitivity (39). For an expected sensitivity of 0.95 (37, 38) with a confidence interval width of 0.1, and assuming an intra-cluster correlation based on the Test Cohort of the study for the previous clearance (40), we estimated a minimum sample size of 17 patients having seizures, for both adult and pediatric patients. No requirement was set for the number of epilepsy patients not experiencing seizures, but we included the available data from all patients to provide the most accurate measure of FAR.

### Reference Standard

The identification of seizures was performed by three board-certified clinical neurologists, who independently examined v-EEG recordings synchronized with the data recordings of the wearable device under evaluation. A “2 out of 3” majority rule inter-rater agreement has been used to mitigate interrater variability in marking v-EEG data for seizure activity (41). The reviewers were blinded to other sources of data, including raw or processed data from the wearable and the algorithm output. Seizure types were classified according to the most recent International League Against Epilepsy (ILAE) seizure classification (42). Two seizure types were targeted in this study: “focal onset to bilateral/unilateral tonic-clonic” (FBTC) seizures, previously known as secondary generalized tonic-clonic seizures, and “generalized onset tonic-clonic” (GTC) seizures, previously defined as primary generalized tonic-clonic seizures. The video-EEG review process consisted of the following steps:

1. The EMU technicians reviewed the v-EEG recordings and filtered out all non-relevant segments. They did not perform any filtering on the remaining v-EEG data. The result is a pruned v-EEG dataset.
2. The research assistants removed any notes in the pruned dataset added by the EMU technician to prevent any potential bias to the three independent reviewers.
3. The principal investigators conducted a review on the pruned v-EEG dataset.
4. Second and third reviewers independently conducted reviews on the pruned v-EEG dataset.

The review process consisted of confirming the onset and offset times of a seizure and assigning a classification label to the event based on the most recent ILAE seizure classification (42). At each site, the following data were documented per patient:

1) v-EEG-based labeled seizure data with clinical observations, and three independent experts validating the labeled events.
2) Seizure onset and end time (video/clinical read).
3) Seizure onset and end time (EEG read).
4) Post-ictal generalized EEG suppression (PGES) duration (if present).
5) Seizure type according to the most recent ILAE seizure classification (42).
6) Clinical presentation.
7) Demographic information (age at enrollment, gender, height, weight, diagnosis, and autonomic related pathologies or relevant chart information).

### Wearable Devices

Two multimodal wrist-worn devices were used in this study, the E4 and the Embrace, both manufactured by Empatica Inc. Both devices received CE medical clearance from the European Union in 2016 (class IIa). Embrace received clearance by the US FDA in January 2018 (Class II) for CS monitoring during periods of rest for adults and in January 2019 for children aged 6 and up.

The E4 wristband embeds a three-axis ACM sensor (sampling frequency: 32 Hz; range; [−2; +2] g), an EDA sensor (sampling frequency: 4 Hz; range; [0.01; 100] μS), a reflective photoplethysmography (PPG) sensor (sampling frequency: 64 Hz), and a temperature sensor (sampling frequency: 4 Hz; range: [−40; +115]°C). The Embrace wristband embeds a three-axis ACM sensor (sampling frequency: 32 Hz; range; [−16; +16] g), a three-axis gyroscope sensor (sampling frequency: 32 Hz; range; [-500; +500] °/s), an EDA sensor (sampling frequency: 4 Hz; range; [0.01; 85] μS) and a temperature sensor (sampling frequency: 1 Hz; range: [−20; +70]°C). The automated detection of CS relies solely on ACM and EDA data. Therefore, only recorded ACM and EDA data acquired by the two devices were used as inputs to the Detection Algorithm. The two devices have been shown to be equivalent in terms of their ACM and EDA sensor data (see details in the Supplementary Material) and therefore were used interchangeably in this study.

### Experimental Protocol

Patients with a known history of epilepsy were admitted for long-term v-EEG monitoring at the EMU of each clinical site. The recruitment process was conducted by each site. Only patients (or their caregivers) who provided their written informed consent were enrolled. All patients were recorded following the same protocol in order to provide homogeneous wearable device data for inclusion in the clinical study.

In all sites, concomitant electrocardiogram (EKG) data were recorded, which were not used either for the seizure labeling nor for informing the classification model of the wearable medical device.

During their time in the EMU, patients wore the E4 or the Embrace wristband, synchronized with the v-EEG at the start of each monitoring period. If seizure semiology reported an asymmetric involvement of arms, the wristband was placed on the wrist where convulsions appeared earlier and/or were more evident; otherwise, the device was worn on the non-dominant arm. When the wearable device used was an Embrace, the patients were also provided with a paired wireless device to download the sensor data from the wearable device and upload them to a dedicated cloud data storage. Following enrollment, study subjects were seen daily during their inpatient hospital stay, continuously monitored with v-EEG and given the usual standard of care.

### Development of the Detection Algorithm

Machine learning algorithms use information embedded in a training dataset, labeled or unlabeled, in order to build a classification model and a decision rule function able to identify and/or distinguish one or more events of interest. The tuning of all the parameters can be performed minimizing a cost function or maximizing one or more performance metrics on validation datasets. The selection of the performance metrics to maximize is usually motivated by the specific application. More specifically, for clinical applications, the performance metrics need to reflect the costs and benefits for patients (43), and thus how to evaluate the performance of a medical device is usually decided at the clinical level (44). The selected performance metrics are described in section Performance Metrics.

**Figure 1:**
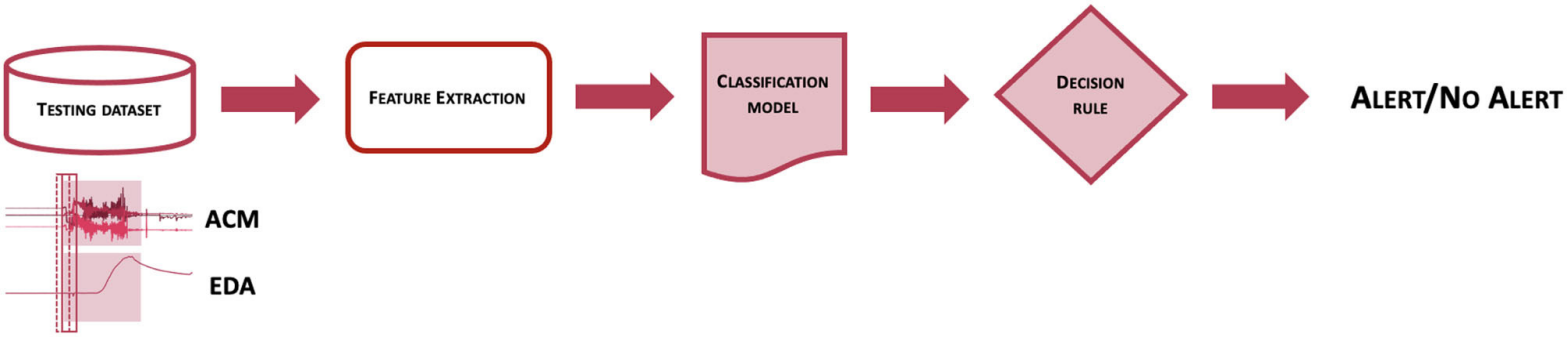
Scheme of the workflow used to classify epochs of EDA and ACM sensor signals into seizure or non-seizure epochs.

Figure 1 represents the workflow of the Detection Algorithm validated in this study. At a very high level, data from the ACM and EDA sensors are processed to compute features from a pre-determined feature set, which are analyzed by a pre-trained classification model to obtain an estimation of the probability that a CS pattern is present in the sensor data. The probability estimates are then evaluated by a decision rule function, to establish whether to issue an alert or not, thus classifying the associated event as a CS.

To identify the features that could represent the pattern of a CS and distinguish it from other types of events, a feature engineering approach was performed. To distinguish CS from all other events, features that characterize both types of events needed to be included in the classification model. A feature set of 160 ACM- and EDA-based features was firstly developed, mostly to better represent the frequency and non-linear characteristics of the sensor data. Due to computational limitations, a subset of 40 features was selected using a sequential floating forward feature selection strategy (45), to maximize the trade-off between performance and computational cost. Features were extracted on consecutive 10-s windows overlapped by 75%.

**Figure 2:**
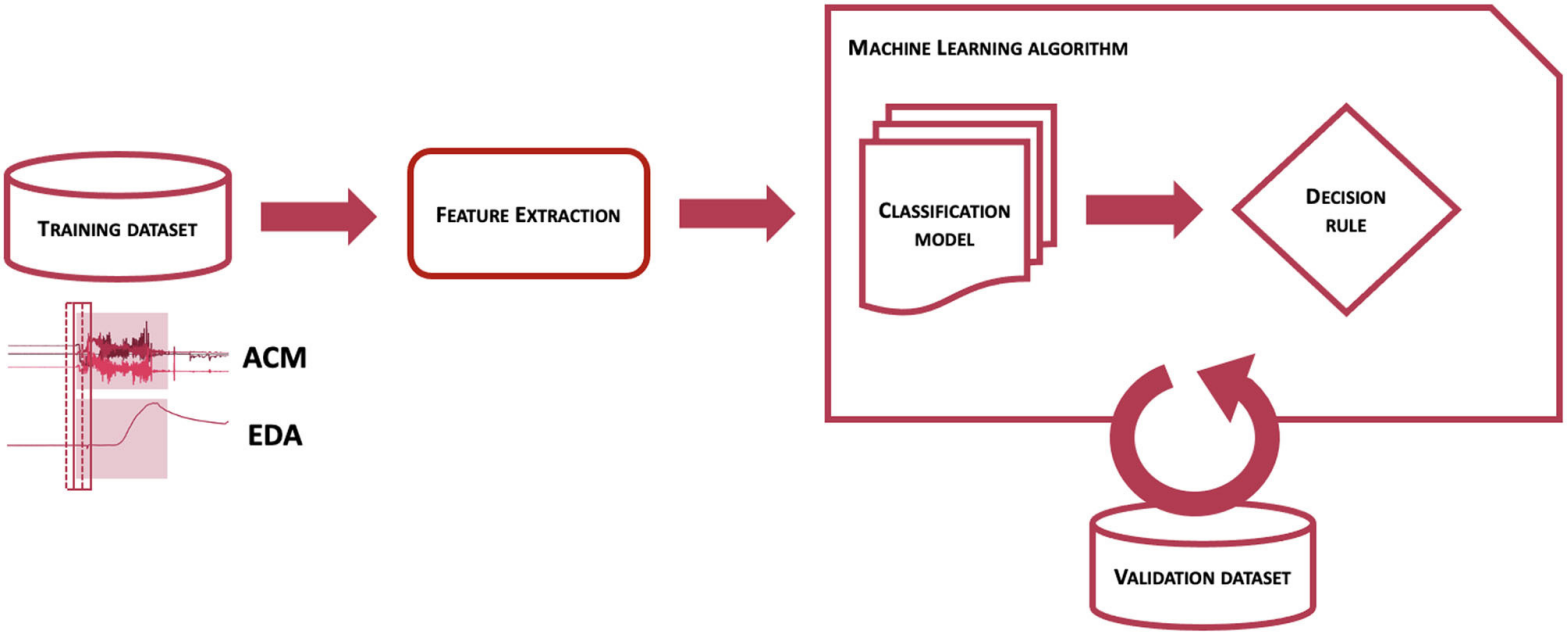
Description of the training phase of the Detection Algorithm.

The process to obtain a classification model for the detection of CS is schematized in Figure 2 and consisted of two main steps. At first, the training dataset, i.e., a collection of labeled sensor data, was processed to obtain a set of features on windowed sensor data. The same procedure was performed on separate validation datasets. Then, after defining performance metrics to maximize, the labeled features from the training dataset were provided to the machine learning algorithm to obtain a classification model and a decision rule function, whose parameters were tuned by maximizing performance metrics evaluated on the validation dataset. To train a classification model able to distinguish CS from non-CS events, it was crucial to provide labeled samples to the machine learning algorithm responsible to build the classification model. For this reason, not only previously recorded clinical data, but also previously logged data from real-life activities showing patterns potentially similar to CS (e.g., tooth brushing, hands clapping, hands washing, gesturing, driving or biking on an uneven surface) were used to make a training dataset, as this procedure of showing both good and bad examples showed improved performance on previous preliminary analyses (46). This process was strictly controlled and highly selective to preserve the correct representability and distribution of the data in the training dataset, to avoid mislabeling of data, and most importantly to prevent overlap between training, validation, and testing datasets. No patients whose data were used in the test sets contributed data to the training or validation processes.

The Test Cohort described in section Test Cohort Allocation and Demographics represents the testing dataset for the Detection Algorithm.

### Rest Detection Algorithm

A proprietary and validated actigraphy-based rest detection algorithm was used to evaluate the performance of the Detection Algorithm during rest conditions (47). Briefly, the magnitude of the 3-axis ACM channels is band-pass filtered. Then, activity counts are obtained as the number of crossings of the ACM magnitude through a specified threshold and accumulated over 30-s epochs. Rest onset and offset are obtained using a rule applied to the moving average of activity counts from a 30-min window. Rest periods <2 h apart were merged assuming a rest interruption between them. The output of the rest detection typically includes sleep periods, and occasionally long quiescent periods of wakefulness.

### Performance Evaluation

#### Performance Metrics

Atrue positive occurred when the Detection Algorithm provided an alarm between the clinical onset and the clinical offset times of an event that was labeled a CS according to the “2 out of 3” majority rule by three independent board-certified clinical neurologists. Given the count of true positives and the expert-labeled number of CS, sensitivity was estimated as the number of true positives divided by the number of expert-labeled CS (“Sensitivity” in **Table 3**). This value was corrected (“cSensitivity” in **Table 3**) for the presence of clusters in the data, more specifically for multiple CS from the same patient, by estimating the intra-cluster-correlation (48) and thus removing the resulting inflation in the sensitivity (39). Similarly, the 95% confidence interval of the so-obtained corrected sensitivity was estimated with the classic Wilson Score method corrected for the cluster effect (39).

A false positive, or a false alarm, occurred when the algorithm provided an alarm not corresponding to any labeled CS. FAR, defined as the number of false alarms per 24 h-worn, a typically reported performance metric in non-EEG seizure detection systems (11), was computed as the total number of false alarms divided by the total recording hours, and normalized for 24 h. The 95% confidence interval of the FAR was computed with a non-parametric bootstrapping method. Specifically, 100,000 samplings with replacement were performed at the level of the patients to incorporate all the sources of within-patient variability (49–51). The number of iterations was chosen equal to 100,000 since it is considered to be a reasonably large number for bootstrapping confidence intervals (49, 52). Since the FAR distribution did not follow a normal distribution, the 95% confidence interval was computed as the 2.5th and the 97.5th percentile (49) of the 100,000 FAR samples.

Two additional statistics were computed to represent a patient-centric point of view on the performance of the detection system: (1) the precision, which is the ratio between the total number of true positives and the total number of alerts, with its 95% confidence interval, computed using the classical Wilson score method (53). In the case of no false alarms (e.g., during rest), to provide a more realistic and conservative estimation for both the precision and its 95% confidence interval, a correction due to Laplace, namely the “Rule of succession,” has been applied, as it has been reported as a good correction for probability equal to 1 with relatively small sample sizes (54); (2) the mean and the standard deviation of the seizure detection latency, defined as the number of seconds between the seizure clinical onset and the algorithm detection time.

To provide a depiction as complete as possible of the relationship between the different performance metrics and the operating points, the receiver operating characteristic (ROC) curve was obtained, which graphs in a two-dimensional space the sensitivity and the false positive rate, or equivalently (1 - specificity), while varying the operating point of the Detection Algorithm (55). For monitoring devices, computing the specificity is not a well-defined task, as while it is easy to count the number of CS events, there are not commensurate “no CS events” that can be easily counted (56). To be able to compute the ROC curve, we assumed that the wearable sensor data periods labeled as non-seizure, could be represented as a sequence of non-overlapping negative events (56), whose duration is equal to the mean duration of the CS events. Additionally, the precision-recall (PR) curve (57) was analyzed, as it is useful when classes are unbalanced, which is the case in epilepsy as most data are from the class “no CS.” The PR curve graphs in a two-dimensional space the recall (an alternative name for the sensitivity) and the precision, while varying the operating curve. It thus attempts to estimate the benefit of detecting the event of interest vs. the burden of providing a false alarm to the patient/caregiver. Finally, the variation of the sensitivity and the FAR at each operating point were analyzed, representing the primary and the secondary endpoints, respectively, for the clinical validation of the detection system. Along with the point estimates of sensitivity, specificity, precision, and FAR, the respective 95% confidence intervals were also computed with the Wilson score method for the proportion-like metrics (sensitivity, specificity and precision) (53), and with a simple normal approximation for the FAR.

All of the components and parameters of the validated wearable device, including all parameters of the algorithm, needed to be “fixed-and-frozen” before the clinical validation. In the Result section, we focus on two operating points of the decision rule function: the first one, FDA-cleared, was fixed under the rationale of maximizing the detection of all the events during periods of rest or low activity; the second one, Active mode, was fixed to balance the ability of the Detection algorithm to identify the majority of the events, while reducing the burden of false alarms on the patients and their caregivers during moderate to intense activities. In section Performance Analysis, the performance metrics are presented for the two different operating points, with a particular emphasis on the FDA-cleared mode. The performance analyses are presented over three groupings of the test data: for all the patients, for pediatric (6–20), and for adult patients (21+). Finally, we present the performance of the seizure detection system during rest, as computed by the automated rest detection algorithm, and show the results for all three groupings.

#### Statistical Tests

Specific statistical tests were performed to establish whether different populations (pediatric vs. adult groups) and behavioral or environmental conditions (rest vs. active groups) showed statistically significant differences. The 95% confidence intervals were computed on each grouping. To test whether there was a statistical difference for “cSensitivity” in each comparison, we computed the 95% confidence interval of the difference between the corrected sensitivities for each group and tested it with a method for independent binomial proportions for clustered data (58). The null hypothesis that there was no difference between the groups can be rejected if the 95% confidence interval does not contain the 0 value. To examine if there were statistically significant differences in the FAR since each group had different exposure times, we performed a normal approximation of a statistical test based on the null hypothesis that the expected number of events experienced by each group were equal (58).

## Results

### Test Cohort Allocation and Demographics

A total of 304 patients' data was recorded from the 6 clinical centers. Upon completion of the study, the Indications for Use (IFU) of the wearable seizure detection device were reviewed by the FDA, which resulted in the exclusion of some patients from the analysis even if the reasons for withdrawal had not been set as exclusion criteria prior to the start of the study. The FDA requested to use only one location for the device, so we chose the wrist. Accordingly, 135 patients were excluded from the study because they did not wear the sensor on the wrist (112)^5^, did not have a prior epilepsy diagnosis (1), or were <6 years old (22), as already explained in Section 2.1. An additional 14 patients were excluded due to hardware, software or data issues of the EEG reference device (10) or the wearable device (4). Only 3 patients were excluded because of lack of compliance (1) or dropping out from the study (2). Table 1 summarizes all of the cases excluded from the study.

**Table 1:** Summary of reasons for withdrawal of patients from the analysis.

**Motivation for exclusion**	**# Patients excluded**	**Description**
Out of the IFU	112	Wearable device placement at the ankle
	22	Age below 6
	1	Non-epileptic seizures
Lost/corrupted data	10	Issues with the reference device (v-EEG)
	3	Corrupted wearable device data
	1	Wearable device hardware failure
Compliance	1	Wearable device not worn
	2	Early termination upon request from the patient

Thus, the analyzed dataset consisted of a total of 152 patients (77 females, age range: 6–63 years, median age: 17 years), of which 85 were pediatric patients (38 females, age range: 6–20 years, median age: 12) and 67 were adults (37 females, age range: 21–63 years, median age: 38 years). The total duration of the recordings was 10,296 h (429 days), including 3,939 h (162 days) from pediatric patients and 6,357 h (265 days) from adult patients. A total of 36 of the 152 patients experienced at least one of the seizures included in the clinical trial during the monitoring period, equally distributed among children (9 females) and adults (6 females). A total of 66 CS events experienced by 36 patients were independently identified by at least 2 out of 3 board-certified clinical neurologists who reviewed the v-EEG recordings: Of these, 35 were experienced by 18 pediatric patients (17 by female pediatric patients), and 31 by 18 adult patients (10 by female adult patients). Table 2 shows the distribution of patients and CS across the different clinical sites, along with the wearable device used by each patient.

**Table 2:** Distribution of patients, wearable device type and CS events across the different clinical sites.

**Site**	**Location**	**Patients**		**Device**		**CS**	
		**Pediatric**	**Adult**	**E4**	**Embrace**	**GTC**	**FBTC**
BCH	US	52 (F: 26)	3 (F: 2)	55	0	5	16
CHOA	US	13 (F: 5)	0	13	0	3	2
EMORY	US	1 (F: 0)	13 (F: 10)	14	0	3	0
NYU	US	0	18 (F: 10)	18	0	0	10
NYUP	US	1 (F: 1)	13 (F: 4)	0	14	0	11
OPBGP	IT	13 (F: 3)	1 (F: 0)	0	14	0	10
RIH	US	5 (F: 4)	19 (F: 12)	24	0	1	5
Total		85 (F: 39)	67 (F: 38)	124	28	12	54

*F, female*.

### Performance Analysis

**Figure 3:**
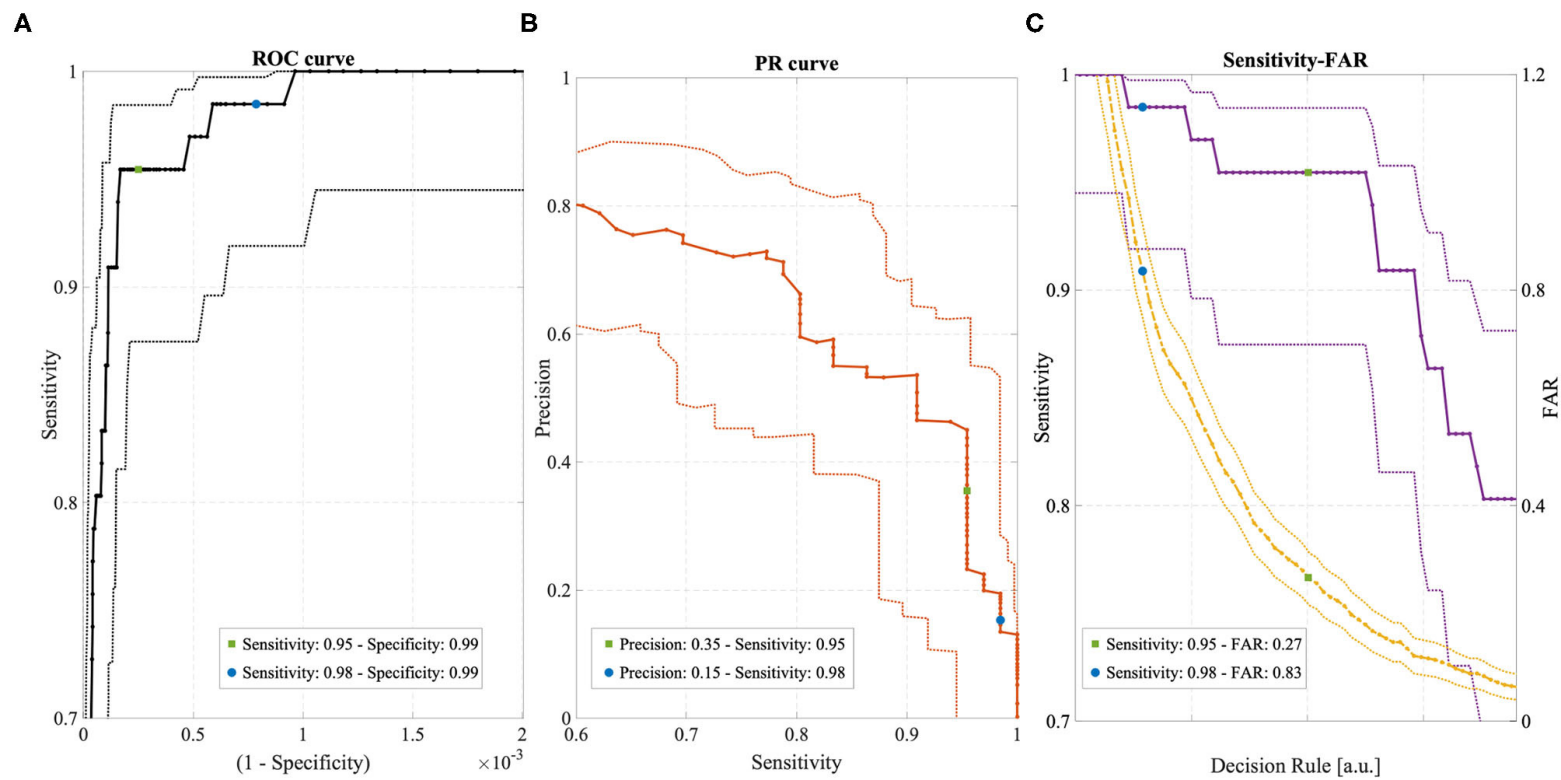
**(A)** ROC curve with 95% confidence intervals (dotted lines). **(B)** Precision-Recall (PR) curve with 95% confidence intervals (dotted lines). **(C)** Sensitivity (violet) and FAR (yellow) curves with 95% confidence intervals (dotted lines). All curves were obtained by varying the operating point of the Detection Algorithm. Two operating points are highlighted in each curve, corresponding to “FDA-cleared” mode (blue circles) and “Active” mode (green circles).

Figure 3 shows the ROC curve, the PR curve and the sensitivity and FAR curves while varying the operating point of the Detection Algorithm. Different portions of the curves are shown to emphasize the two selected operating points and their relationship with the different performance metrics. The specificity spanned a very narrow range of values, very close to 0.99, due to the highly unbalanced class distribution and the high specificity of the Detection Algorithm. The sensitivity at the two operating points was 0.95 and 0.98, respectively, with a higher value for the FDA-cleared operating point (blue circle in Figure 3). The overall precision across all conditions, rest and non-rest, was relatively low: for the FDA-cleared operating point the precision was 0.15, which indicates 1 true detection for every 6 false alerts on average, while for the Active operating point the precision was 0.35, resulting in 1 true detection for every 2 false alerts. The FAR of 0.84 implied on average <1 false alarm per full day of recording at the FDA-cleared high-sensitivity operating point, and the FAR of 0.27 implied on average about 1 false alarm every 4 days of continuous wear while operating in the less sensitive Active mode.

**Figure 4:**
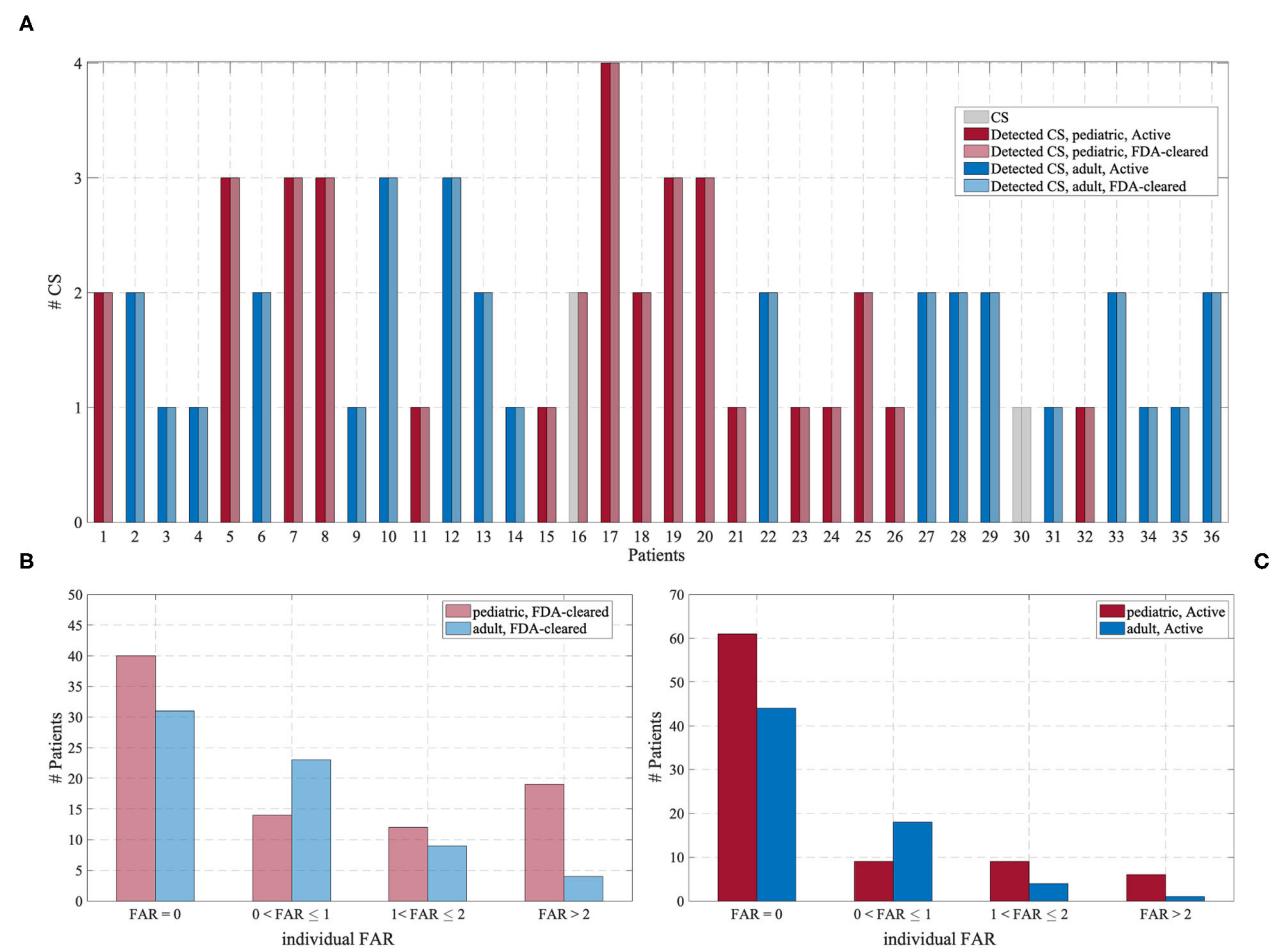
**(A)** Number of CS (gray) and the number of detected CS for each of the 36 patients experiencing CS, grouped according to the operating point of the Detection Algorithm and the age group. **(B)** Distribution of the individual FAR for the “FDA-cleared” mode. **(C)** Distribution of the individual FAR for the “Active” mode.

Figure 4 shows the detected CS and the FAR for each patient in the Test Cohort, grouped according to the operating point of the Detection Algorithm and the age group. In the Active mode, most patients experienced no false alarms (72 and 63% for pediatric and adult patients, respectively, had FAR = 0). In the FDA-cleared mode, an individual FAR of 0 was experienced by 47 and 46% for pediatric and adult patients, respectively.

Table 3 reports the characteristics of the datasets included in each performance evaluation (rows “Test cohort”) and the results for each operating point (rows “FDA-cleared” mode and “Active” mode), for the whole Test Cohort (columns “Overall”) and each age group separately (columns “Pediatric” and “Adult,” respectively). Also, the performance evaluation was conducted including all the data (columns “All data”), or only the data recognized as rest by the automated rest detection algorithm described in paragraph 2.7 (columns “Automatically-Detected Rest”). Overall, rest periods accounted for ~38% of the total recording duration. The median duration of rest periods was 7.1 h and the median number of rest periods per recording day was 1.2. These statistics lend support to a hypothesis that most of the rest periods were probably sleep periods. More detailed statistics are reported in the Supplementary Figure 2.

**Table 3:** Characteristics of the Test Cohort and performance of the Detection Algorithm for all the patients and the two age groups, for the two different operating points of the algorithm (“FDA-cleared” mode and “Active” mode, and for all the data (“All data”) vs. only periods of rest (“Automatically-Detected Rest”).

		**All data**			**Automatically-detected rest**		
		**Overall**	**Pediatric**	**Adult**	**Overall**	**Pediatric**	**Adult**
Test cohort	# Patients (w/ CS)	152 (36)	85 (18)	67 (18)	144 (13)	78 (7)	66 (6)
	Total hours	10,296	3,939	6,357	3,995	1,703	2,292
	Average hours/pat.	68	46	95	26	20	34
	Total CS	66	35	31	20	13	7
	Mean duration [sec] (St. Dev.)	84.56 (34.57)	89.37 (39.09)	79.13 (28.27)	78.65 (26.49)	79.61 (24.25)	76.86 (32.26)
“FDA-cleared” mode	Detected CS	65	34	31	19	12	7
	Mean delay [sec] (St. Dev.)	37.46 (21.09)	37.76 (24.06)	37.13 (17.64)	33.05 (12.09)	32.08 (7.77)	34.71 (17.96)
	Sensitivity	0.98	0.97	1.00	0.90	0.92	1.00
	cSensitivity [95% CI]	0.96 [0.92, 1.00]	0.92 [0.85, 1.00]	0.94 [0.89, 1.00]	0.83 [0.69, 0.97]	0.82 [0.65, 0.99]	0.82 [0.65, 1.00]
	Precision [95% CI]	0.15 [0.12, 0.19]	0.14 [0.10, 0.19]	0.17 [0.12, 0.23]	0.95* [0.76, 0.99]	0.93* [0.66, 0.99]	0.89* [0.52, 0.98]
	Total false alarms	357	207	150	0	0	0
	FAR [95% CI]	0.83 [0.63, 1.07]	1.26 [0.87, 1.73]	0.57 [0.36, 0.81]	0	0	0
“Active” mode	Detected CS	63	32	31	19	12	7
	Mean Delay [sec] (St. Dev.)	40.03 (16.25)	37.44 (12.96)	42.71 (18.91)	38.26 (12.43)	36.75 (7.62)	40.86 (18.57)
	Sensitivity	0.95	0.91	1.00	0.90	0.92	1.00
	cSensitivity [95% CI]	0.91 [0.84, 0.99]	0.85 [0.72, 0.98]	0.94 [0.89, 1.00]	0.83 [0.69, 0.97]	0.82 [0.65, 0.99]	0.82 [0.65, 1.00]
	Precision [95% CI]	0.36 [0.29, 0.43]	0.33 [0.24, 0.43]	0.39 [0.29, 0.50]	0.95* [0.76, 0.99]	0.93* [0.66, 0.99]	0.89* [0.52, 0.98]
	Total false alarms	113	65	48	0	0	0
	FAR [95% CI]	0.27 [0.18, 0.36]	0.40 [0.23, 0.59]	0.18 [0.10, 0.28]	0	0	0

CI, Confidence Interval. *Laplace correction (i.e., the “rule of succession”) was applied to improve the estimation of the precision and its 95% confidence interval.

As expected, the estimated sensitivity was inflated by the presence of clustered data (i.e., multiple CS per patient), and therefore we computed its statistical correction “cSensitivity,” which was lower. Nonetheless, the lower bound of the 95% confidence interval for cSensitivity was higher than 0.7 for all age groups and both operating points when considering the whole dataset in the analysis (columns “All data”). Even though only one operating point has been clinically validated and FDA-cleared, the less sensitive operating point (“Active” mode, in Table 3) also reached a good overall performance in detecting CS.

The requirement expressed in the secondary endpoint regarding the FAR has been met for all three age groupings and both operating points. When considering only the data recognized as rest by the rest detection algorithm, corresponding to sleep periods and occasional long periods of inactivity, the number of the false alarms dropped to 0 for all the grouped analyses and both operating points, leading ideally to a FAR equal to 0. As a consequence, the precision drastically increased. Even applying a conservative estimate of precision with a 95% confidence interval adjusted by the “rule of succession” correction of Laplace, the corrected precision reached a value of 0.95.

Table 4 shows the results of the statistical analysis of the main performance metrics, i.e., sensitivity and FAR, between the age groups and the activity groups. As expected, there was no difference in the ability of the Detection Algorithm in identifying CS between the two age groups and between the two contexts in which the CS occurred, i.e., during rest or during a moderate to high activity. On the contrary, the difference in the occurrence of the false alarms was statistically significant, as expected when comparing rest vs. moderate to high activity, and between the two age groups.

**Table 4:** Statistical comparison of “cSensitivity” and FAR between pediatric and adult patients, and between Rest and Active periods, for the two operating points.

**Operating point**	**Variable**	**Groups**	***p*-value**
“FDA-cleared”	cSensitivity	Children vs. adults	0.177
		Rest vs. active	0.496
	FAR ratio	Children vs. adults	** < < ** **10** ^**−3**^
		Rest vs. active	** < < ** **10** ^**−3**^
“Active”	cSensitivity	Children vs. adults	0.478
		Rest vs. active	0.227
	FAR ratio	Children vs. adults	**<** **10** ^**−3**^
		Rest vs. active	** < < ** **10** ^**−3**^

*Values in bold indicate a statistically significant difference (p-value < 0.05)*.

The detection latency of the system was on the order of 30–40 s (Table 3). Seizures occurring during the whole recording period were detected with a median detection latency of 37.46 s and 40.03 s when using the FDA-cleared or the Active mode, respectively. Considering only seizures occurring during rest periods, the latency was 33.05 s and 38.36 s with the two modalities, respectively.

## Discussion

### Key Findings and Advances Over Prior Research

Wearable technologies designed to accurately and automatically monitor for CS seizures provide advantages of improved detection and alerting to caregivers of potentially life-threatening events, enabling attention to seizures, and potentially lowering the risk of serious injury or death from accidents and SUDEP. A recent study of 255 SUDEP cases (definite and probable) and 1,148 matched controls showed that 69% of SUDEP cases in patients with GTC seizures who live alone may be prevented if patients are attended, or if their GTC seizures are controlled. Recent practical clinical guidelines recommend using clinically validated devices for automated detection of CS, the seizure types included in this study, especially in unsupervised patients, where alarms can facilitate rapid interventions (28).

To the authors' knowledge, this is the first prospective study on a multimodal wearable CS detection system based on wrist ACM and EDA sensors evaluated on a large patients' pool (152 patients). Prior work has presented prospective analyses of non-EEG seizure detection devices (31–35), but none of them combined ACM and EDA sensors or used multi-modal methods on continuous 24-h patient data, i.e., including activity as well as sleep. While ACM sensors are intuitively fundamental to capture signs of ongoing CS, EDA sensors, which convey information on sympathetic autonomic nervous system activity, improve the specificity of the detection (37) and provide additional information for seizure characterization (59, 60). Apart from the unique combination of sensors to the authors' knowledge, the presented Detection Algorithm is the only machine learning algorithm used in commercialized non-EEG seizure detection systems. Machine learning algorithms are becoming increasingly recognized as effective tools for the detection of seizures (61, 62), despite the challenges they pose for traditional medical regulatory systems (63).

This work further contributes to the field detailed analyses examining performance differences between pediatric and adult patients, between rest and active conditions, and using two different operating modes of the automated algorithm (both defined *a priori* during the previous training phase of the Detection Algorithm and fixed and frozen before applying them to the test data here). To the authors' knowledge, these types of analyses are novel and provide an expanded understanding of the capabilities and potential shortcoming of the wearable multimodal system under investigation.

This study may qualify for the recently proposed label of a phase III validation study (28, 29): Multiple EMU centers were involved; the reference standard was v-EEG recordings interpreted by experts; more than 20 patients (*n* = 36) with seizures were included with more than 30 seizures (*n* = 66); the data and patients analyzed were disjointed from those used to develop the Detection Algorithm, removing the risk of overfitting, and all of the analyses were performed in a real-time manner fully mimicking the functioning of the algorithm on-board. Offline analysis of bio-signals may raise the possibility of overfitting to the recorded data set and can call the generalizability of results into question (28). However, given that the Detection Algorithm was trained on separate data and a fully separate patient group, and that it was “fixed-and-frozen” before being applied to the test set, and still uses the same code that runs on-board the Embrace device, we believe that overfitting is not affecting these results, as also supported by FDA's careful evaluation.

The performance of the FDA-cleared Detection Algorithm complies with and surpasses the performance requirements on non-EEG seizure monitoring devices, which focus on sensitivity and FAR. The Detection Algorithm showed an excellent sensitivity, capturing 65 out of 66 CS occurred in 36 patients, with a lower bound of the 95% confidence interval substantially higher than the study endpoint on sensitivity. The system provided reasonably timely detection of CS, within an average of 37.46 s from the onset of clinical manifestations as annotated by expert v-EEG raters. Rapid detection is of utmost importance, given that timely treatment of seizures can be life-saving, especially after CS, which bear a higher risk of SUDEP (3, 13). Even if the observed delay is slightly higher than systems using arm-worn electromyography patches (33, 35), it is comparable to previous results using wrist-worn ACM-only sensors (31) and combined ACM and EDA sensors (38) which seem to be preferred by patients (19). A delay of ~30–40 s seemingly would be sufficient warning to allow caregivers to provide interventions (if they are nearby), e.g., turning patients to minimize postictal respiratory dysfunction, considering that the minimum duration of CS is around 30–40 s (64, 65) and that apnea, bradycardia and oxygen desaturation onset may occur in the postictal phase, ~50–150 s after the onset of GTCS (66).

The FAR was well-below the endpoint of <2 false alarms per day, with almost half of the patients experiencing no false alarms and only 15% of all the patients experiencing more than 2 false alarms per day. The precision of the Detection Algorithm was relatively low (~0.15) indicating that around 1 out of 7 alerts was a true seizure; this is an area where continued improvement is needed. There is a very large variance in the prevalence of CS (67), resulting in a very large range of individual precision estimates; thus, the ratio of true alarms to false ones can vary widely (4, 68). This may be a reason why FDA does not include precision, but focuses on sensitivity and FAR for evaluating non-EEG seizures detection systems (29).

The FAR provides a measure of the average frequency of false alarms independently from the frequency of the CS individually experienced by the patients, resulting in an estimate of the potential burden of false alarms in the daily life of the patients and their caregivers.

When comparing the present results with the previous phase II multi-center study using the same sensor set (38), the Sensitivity was slightly higher (0.96 with CI = [0.92, 1.00] in the current study vs. 0.94 with CI = 0.85–0.98). The FAR observed in the previous study when pooling data from the 69 patients was lower (0.2) than the FAR observed in this study (0.83) on all 152 patients. This difference might be ascribed to the much longer duration of the recordings in the present study (429 days vs. 247 days), likely containing more varied motor patterns during longer awake time^6^ and a higher absolute and relative number of more active, pediatric patients in the present study (85/152 vs. 24/69 patients). The prior phase III validation of another multimodal seizure detection device based on ACM and PPG (32) reported a Sensitivity of 0.96 (CI = 0.8–1.00) on 22 tonic-clonic seizures and a median FAR of 0.25 per night (CI = 0.04–0.35). However, the system was tested only during nighttime and only on a group of patients that included some of the same people used to develop the algorithm, two conditions that can inflate the algorithm's performance. The present results, both sensitivity and FAR, show improvements over the FDA-pivotal study for a surface-EMG bicep-worn automated seizure detection system (SPEAC; Brain Sentinel), which originally detected 35 of 46 GTC seizures (0.76 with CI = 0.61–0.87) with a FAR of 2.52 per 24 h, and with corrected midline-biceps positioning was improved to detect 29 of 29 GTC seizures (1.00, CI = 0.88–1.00) with a mean FAR of 1.44 per 24 h (35). Another phase III study on a surface-EMG bicep-worn device (EDDI; Ictal Care) reported a sensitivity of 0.938 (30 out of 32 GTC seizures were detected, CI = 0.86–1) with a mean FAR 0.67 per 24 h (33), slightly lower than the FAR observed here but evaluated on much shorter periods (155 days from 71 patients). The present results also showed sensitivity improvements over a previously published phase III study of a wrist-worn ACM-triggered seizure detector (Epi-Care; Danish Care Technology, Sorø, Denmark) evaluated in EMU's that showed a sensitivity of 0.9 (CI = 0.85–1.00) and a FAR of 0.2/day for detecting bilateral tonic-clonic seizures (31). The same device was later evaluated in what was described as a phase IV field study (34), again reporting a median sensitivity of 90% but with a lower average FAR of 0.1/day. Of the patients who completed the latter study (ages 7–72, average = 27), about half were in an institution, 27% used it only at night, and four patients discontinued use because of a high FAR. The use only at night and the removal of participants having a high FAR are adjustments that we did not make in our study, which make the two sets of results less comparable as each of these adjustments generally reduces the FAR. That ambulatory study differs from our study also in that its seizure logs were based on observation, without v-EEG confirmation; these factors raise the possibility that seizures might have been missed both by the device and by human observers. The prospective nature of the present study as well as its longer duration of recordings and validated labels (with both seizure and non-seizure epochs validated separately by three independent experts using only video and EEG while blinded to the wearable data) make it more valuable in terms of providing realistic gold-standard performance estimates.

The comparison between pediatric and adult patients did not show significant differences regarding Sensitivity. The only missed CS, when using the FDA-cleared Detection algorithm, was from a pediatric patient whose convulsions were rather mild (by inspection of the ACM sensors). Our findings are in line with the absence of difference between pediatric and adult seizures reported in the literature. The seizure types used in this work are independent of patient characteristics such as age and gender. In the 11 classifications of epileptic seizures and epilepsy syndromes and revisions by the ILAE, starting in 1964 and ending in 2017 (69), no distinction has been made for tonic-clonic seizures in patients of different gender or age. Very few studies have been published about the differences by age or gender in the EEG or clinical features of CS, and in none of the seizure types we examined has age or gender been identified as a significant factor of differentiation (70–72). Moreover, in the non-EEG-based seizure detection literature, a pivotal trial that was used to clear a motion-based CS detection device for medical use in Europe presented no distinctions in age or gender of patients (31). Differently from the sensitivity, a significant difference for the FAR between the two age groups was observed, even if the performance was in line with the recommended limits (FAR <2) for both subgroups. This may be ascribed to the fact that children are more likely than adults to engage in repetitive, activating motions (like excitedly shaking a dice, dancing, or playing video games, etc.) while in the inpatient EMU, which resulted in a higher number of false alerts. It is worth noticing the high variability of the individual FAR perceived by the patients during their admission in the EMU. Counterintuitively, pediatric patients more frequently experienced no false alerts than adults, but at the same time pediatric patients experienced overall more false alerts than adults. Specifically, pediatric patients with FAR higher than 2 outnumbered adult patients. In other words, a few pediatric patients had a very high FAR, which raised the group average FAR.

The comparison between periods of rest and periods of activity showed that the FAR was significantly higher during periods of activity, for both age groups. This was not surprising, as non- seizure motor patterns resembling convulsions (e.g., periodic movements with relatively high frequency) are more likely to happen during periods of activity. During sleep, the number of false alarms was 0, while all seizures except one were correctly recognized. Issuing an alert for real seizures during sleep is fundamental to mitigate the risk of SUDEP (73). Having a precision of 100% during sleep is important to reduce the burden on both caregivers and patients.

To provide a detailed overview on the capability of the Detection Algorithm, results were presented at two different operating points: Active mode, designed to be less sensitive but more specific than FDA-cleared mode, is characterized by a FAR 68% lower than the FDA-cleared one, while keeping the sensitivity slightly lower than the FDA-cleared mode, and most importantly still above the requirement. At a first glance, the advantage of FDA-cleared mode vs. Active mode (i.e., a higher sensitivity) doesn't seem to counterbalance the cost of an increase in the FAR. However, it's worth considering that for applications developed for saving lives, or to prevent serious consequences, as the case of the Detection Algorithm presented here, the cost of type II errors (missed events) is higher than the cost of type I errors (false positives). For the most dangerous context—sleep—the FAR is equivalent (FAR=0 for both operating points), so identifying more CS events becomes the key discriminating factor for the selection of the operating point. Finally, the decrease in the detection time, mostly during sleep, is another important factor that suggests operating the Detection Algorithm at its more sensitive configuration: a timely intervention in the case of a near-SUDEP can dramatically increase the chances to save the patient's life (74). The Embrace system currently allows the user to switch between the FDA-cleared mode and the Active mode, which is suggested during situations in which a lower FAR is desirable. For example, the patient may switch to Active mode when engaging in daytime activities likely to cause false alerts and when they can be sure that their chances of a CS are relatively low, as some patients have certain times of the day or certain phases of a hormonal or other multidien cycles (75) with very low seizure probability.

### Limitations

One of the significant strengths of this study is also its most significant limitation: the system has been tested in EMU environments. Its validation for outpatient environments still needs to be fully documented via an appropriately large “phase IV” study, following the recommendations of the scientific community (28, 29). To those recommendations, we have also suggested to add additional criteria that we think are important such as making sure no participants in the test set were used to develop or tune the algorithm, and making sure that there is a high-quality process in place to validate both the presence *and absence* of any seizures in the field, as there is typically no video or EEG when seizures happen in daily-life outpatient settings. Outpatient settings typically involve increased patient movement, which as we saw in the EMU was correlated with higher FAR. In an outpatient setting, if a seizure happens when a patient is alone and a device (with poor sensitivity) does not alert anybody to come, then the seizure may not be noted in a diary and it may not be properly counted as a “missed event.” Patients are well-known to underreport CS and thus if the patient is not continuously observed, this can result in a reported sensitivity that is significantly inflated, as the number of undetected CS will be under-reported (14). Preliminary studies, where reliable observers accompanied outpatients continuously to label their data, have shown that the performance of a previous version of the ACM and EDA Detection Algorithm, when evaluated in outpatient settings, has been comparable to the performance in inpatient settings in both short-term and explorative longitudinal analysis (36).

### Future Research Directions

Future research goals include further reducing the FAR without reducing the sensitivity of the Detection Algorithm. Future goals also include adding additional modalities to the ACM and EDA to discriminate between epileptic and non-epileptic events (76) and to detect other types of motor epileptic seizures, such as myoclonic seizures [for which a preliminary analysis showed promising results (77)]. The recognition of non-convulsive seizures, e.g., focal seizures, is also a target of growing interest. At present, a clear evidence gap has still to be filled before introducing the automated ambulatory detection of non-convulsive seizures into clinical practice (28, 78, 79). However, promising results using the E4 wristband indicated that this may be possible with a wrist-worn device (80–82). Additionally, advanced post-processing analytics on the peri-ictal periods may provide seizure semiology information, thereby expanding the quality of available patient data. The characterization of the post-ictal phase may also be useful to determine the risk of SUDEP (83); the wearable sensor studied here continuously monitored activity vs. inactivity, sleep/wake, respiration during rest, and sympathetic nervous system function at the time of a recorded “probable SUDEP” where an alert was sent but nobody arrived, and a large surge in EDA occurred (27). Several biomarkers of interest in SUDEP, in seizure-prevention, and other neurological studies can be monitored continuously by a smart watch, particularly if it also measures EDA (59, 60, 84). The development of automated methods for objective risk assessment of the recorded seizures may lead ultimately to a paradigm shift of patient monitoring and outcome assessment in the field of mobile seizure detection (22).

## Data Availability Statement

The datasets presented in this article are not readily available because the sensor data recorded on the patients are proprietary of Empatica Inc. The authors can share the data about seizure counts and the output of the Detection Algorithm for each patient upon request to the corresponding author. Requests to access the datasets should be directed to francesco.onorati.bio@gmail.com.

## Ethics Statement

The studies involving human participants were reviewed and approved by the New England Independent Review Board (WIRB-Copernicus), Needham, MA, US, and the Ethics Committee of Bambino Gesù Children's Hospital, Rome, Italy. Written informed consent to participate in this study was provided by the participants' legal guardian/next of kin.

## Author Contributions

FO, GR, and RP contributed to the conception and design of the study. WL, AB, JB, PD, RE, TL, FM-T, RS, DF, and JJ contributed to data collection and the labeling of v-EEG data. CC organized the database. FO and GR defined the performance and statistical analysis presented in the paper. FO performed the analysis reported in the Results section. FO and GR wrote the first draft of the manuscript. RP substantially contributed to the revision of the manuscript. All authors contributed to the article and approved the submitted version.

## Conflict of Interest

FO, GR, and CC are shareholders of Empatica Inc., which manufactured two of the devices used in this work and developed the two new algorithms tested in this work. GR is also an employee of Empatica and RP is also a consultant and chairs the board of directors for Empatica. TL is part of pending patent applications to detect and predict seizures and to diagnose epilepsy with devices different from the ones used in this work and has received research support from Empatica to conduct this research. TL, WL, and AB have received sensors from Empatica to perform the reported research. The remaining authors declare that the research was conducted in the absence of any commercial or financial relationships that could be construed as a potential conflict of interest.

## Publisher's Note

All claims expressed in this article are solely those of the authors and do not necessarily represent those of their affiliated organizations, or those of the publisher, the editors and the reviewers. Any product that may be evaluated in this article, or claim that may be made by its manufacturer, is not guaranteed or endorsed by the publisher.

## Footnotes

^1^Note that the FDA does not declare a wearable device to be “approved”; the language they use after validating a wearable medical device and accepting its claims to allow it to be marketed is that the device is “cleared”.

^2^Advanced epilepsy center is equivalent to a Level IV center in US.

^3^The abbreviation “NYUP” (i.e., NYU Pivotal) differentiates data recorded for the pivotal study from data previously recorded at the same medical center. Different patients have been enrolled in the new data collection with respect to the previous study. The two studies were performed at different times and using two different wearable devices (Empatica E4 for NYU and Empatica Embrace for NYUP).

^4^“Generalized Seizure Detection And Alerting In The EMU With The Empatica Embrace Watch And Smartphone-Based Alert System” (ClinicalTrials.gov Identifier: NCT03207685).

^5^Some patients, especially with sensory disorders, may become distracted or stressed by a wrist-worn device. Some patients had wrists that were too small for the E4 device. To allow both types of patients to contribute data, they could wear an E4 on the lower calf, just above the ankle. However, FDA later asked us to focus only on data from one location, so we chose the wrist.

^6^During longer-duration recording periods it is more likely that the patients are not always resting, but are getting up and about, engaging in more diverse activities that trigger more false alarms; thus, the estimation of the FAR when made over longer durations may be higher, even though it is always normalized by the duration.

## Supplementary Material

Supplementary Figure 1 Timeline of the data collection at each clinical site.

Supplementary Figure 2 Statistics on rest periods. **(A)** Distribution of the duration of the detected rest periods. **(B)** Distribution of the individual number of rest periods per days of recording.
