# Supplementary figures and images for: Prospective Study of a Multimodal Convulsive Seizure Detection Wearable System on Pediatric and Adult Patients in the Epilepsy Monitoring Unit
Source: Front Neurol. 2021 Aug 18;12:724904. doi: 10.3389/fneur.2021.724904 (PMC8418082; doi:10.3389/fneur.2021.724904)

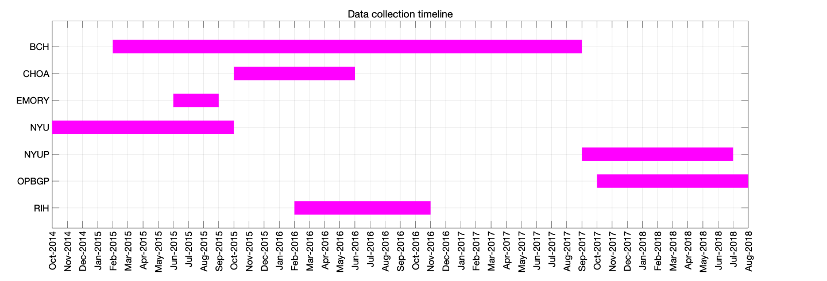

Supplement: Supplementary Figure 1 — Timeline of the data collection at each clinical site. [file Image_1.TIF]

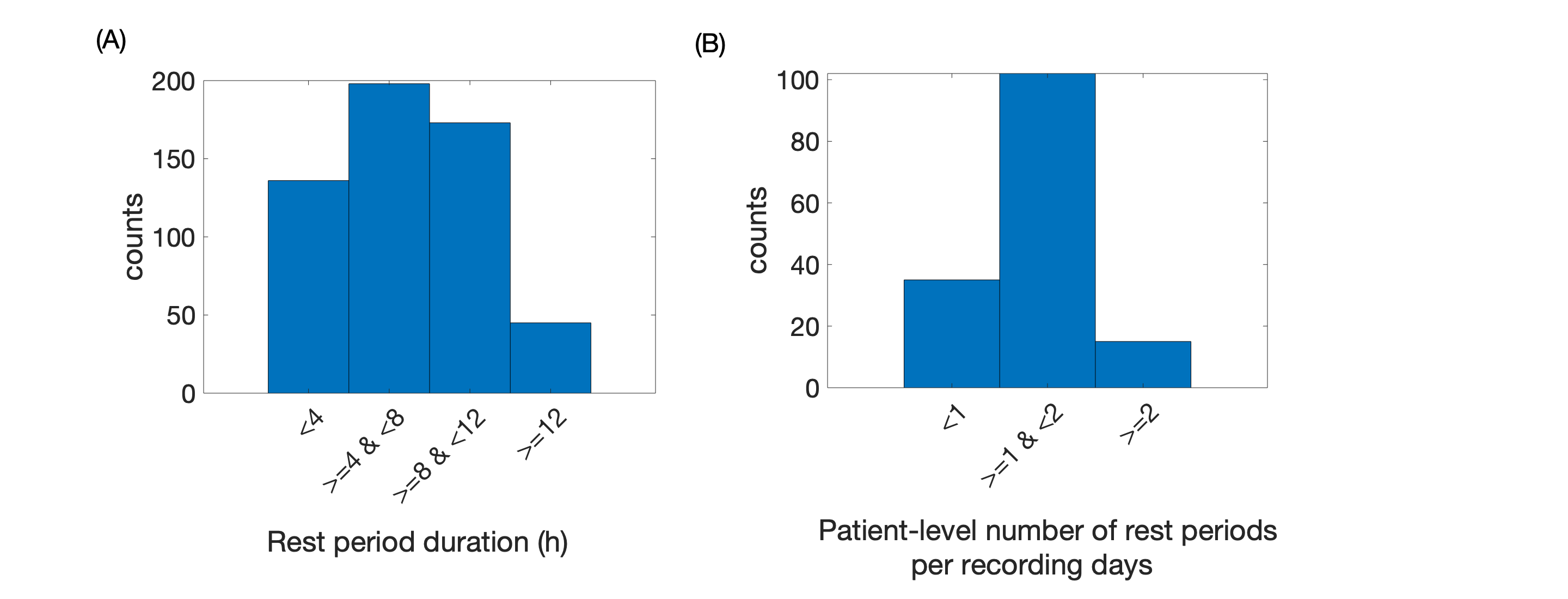

Supplement: Supplementary Figure 2 — Statistics on rest periods. (A) Distribution of the duration of the detected rest periods. (B) Distribution of the individual number of rest periods per days of recording. [file Image_2.TIF]
