# Supplementary material for: Prospective Study of a Multimodal Convulsive Seizure Detection Wearable System on Pediatric and Adult Patients in the Epilepsy Monitoring Unit
Source: Front Neurol. 2021 Aug 18;12:724904. doi: 10.3389/fneur.2021.724904 (PMC8418082; doi:10.3389/fneur.2021.724904)
Supplement: Supplementary file 3 [file Table_1.DOCX]

**Comparison between Embrace and E4 Accelerometry and Electrodermal Activity sensors**

This document reports the results of bench tests performed to compare the Empatica E4 and the Empatica Embrace devices in terms of Accelerometry (ACC) and Electrodermal Activity (EDA) sensors. The sensor readings have been compared by means of absolute error, accuracy and precision (*ISO 5725-1, 1994*). The absolute error is the median of the absolute errors between the measured values and the real value, expressed as measurement units. The accuracy is the median of the absolute error between the measured values and the real value, expressed as percentage with respect to the real value. The lower the accuracy, the closer the measured value is to the real value. The precision is the random spread of the measured values, expressed as interquartile range of the readings in measurement units. The lower the precision, the more repeatable the measurement is.

1. **Comparison of EDA sensor data**

**1.1 Methods**

To compare EDA measurements performed by Embrace and E4, laboratory tests were conducted by sequentially connecting the inputs of the respective EDA circuits to off-the-shelf electrical resistors. Devices’ outputs were compared to the output of a digital multimeter (Fluke, accuracy=1%), used as a reference device. The chosen resistances (i.e., conductance 1 [eda1] = 11.4 𝜇S and conductance 2 [eda2] = 47.6 𝜇S) lie within the range of typical skin conductance levels that can be measured on the wrist (*Poh et al, 2012; Sarkis et al, 2015; Picard et al, 2015*). For both E4 and Embrace, tests were performed on 17 different devices at room temperature.

**1.2 Results**

Values measured by E4 and Embrace are shown by the box-plots of Figure 1 (left panel: 11.4 𝜇S; right panel: 47.6 𝜇S). Each point represents the average of the EDA circuit output over a 5 seconds acquisition.

The plots are rescaled to zoom in on the error. However, all samples lie within the accuracy range of the reference device (i.e., black dashed lines) which is 1% of its measurement.

**
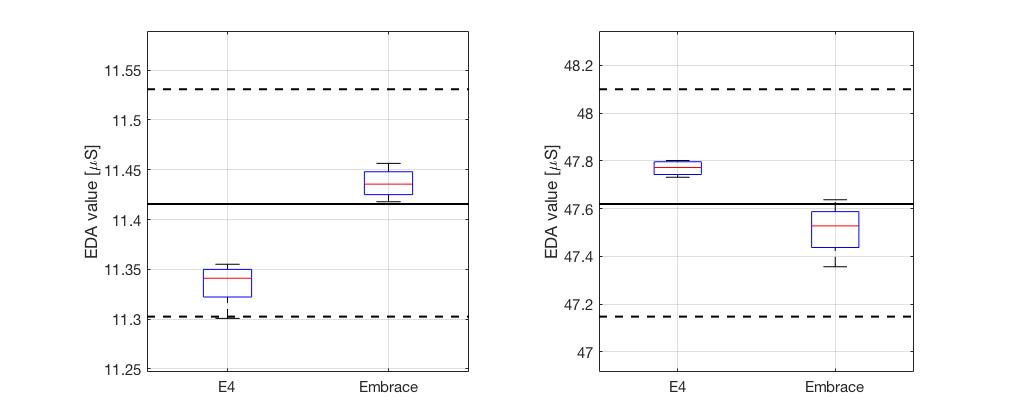
**

**Figure 1. Left panel:** Box plot of EDA values measured by E4 and Embrace (n=17) at a nominal conductance input of 11.4 𝜇S. **Right panel:** Box plot of EDA values measured by E4 and Embrace (n=17) at a nominal conductance input of 47.6 𝜇S. Black horizontal lines in the two plots represent the outputs of the reference device, while black dashed lines represent the accuracy range of the reference device making these measures.

Table 1 summarizes the absolute error values (𝜇S), precision (𝜇S) and the accuracy (%) (i.e., absolute error divided by the value of the reference device) of each device with respect to the reference measurement, for each level of conductance.

|  | **Absolute error E4 (𝜇S)** | **Absolute error Embrace (𝜇S)** | **Precision E4 (𝜇S)** | **Precision Embrace (𝜇S)** | **Accuracy E4 (%)** | **Accuracy Embrace (%)** |
| --- | --- | --- | --- | --- | --- | --- |
| **Eda level 1** | 0.07 | 0.02 | 0.03 | 0.02 | 0.65 | 0.18 |
| **Eda level 2** | 0.15 | 0.09 | 0.05 | 0.15 | 0.32 | 0.19 |

**Table 1.** The first four columns represent the absolute error (𝜇S), expressed as median, and the precision (𝜇S), expressed as interquartile range, of the E4 and Embrace at the two different levels of conductance. The last two columns show the accuracy, the median of the absolute error between the measured values from E4 and Embrace and the real value, expressed as a percentage with respect to the real value.

- 1. **Conclusions**

While the Embrace EDA circuit provides a finer (improved) resolution (i.e., lower 𝜇S/LSB reported in Table 1) than the E4, the resolution of both devices is low enough to comply with standard requirements for EDA measurements (*Bouscein, 2012*). Outputs from both devices are in agreement with the reference device, with absolute errors in the same order of magnitude. For both devices, accuracy (as a measure of error) is lower than 1% with comparable precision.

1. **Comparison of ACC sensor data**

**2.1 Methods**

To compare ACC sensor data from Embrace and E4, both devices were placed on a test jig programmed to generate predetermined motion profiles. For static tests, the devices were simultaneously moved in 3 different static positions, each one held for 3 seconds. For dynamic tests, the devices were simultaneously subjected to a uniform, simple harmonic motion produced by a DC motor rotating at a speed of 4 rad/s. Three-axis ACC sensors data were recorded from 4 pairs of Embrace and E4 and acquired at a sampling rate of 32 Hz.

**2.2 Results**

**2.2.1 Static tests**

Figures 2, 3, and 4 show the results of the static position test for the accelerometer X, Y, and Z axis, respectively. In each figure, the top graph represents the acceleration measured by the sensors (expressed in g-units) for the 8 tested devices, while the bottom graphs depict the box plot of the samples acquired during the 3 static positions (excluding the transition periods), for both E4 and Embrace.

**
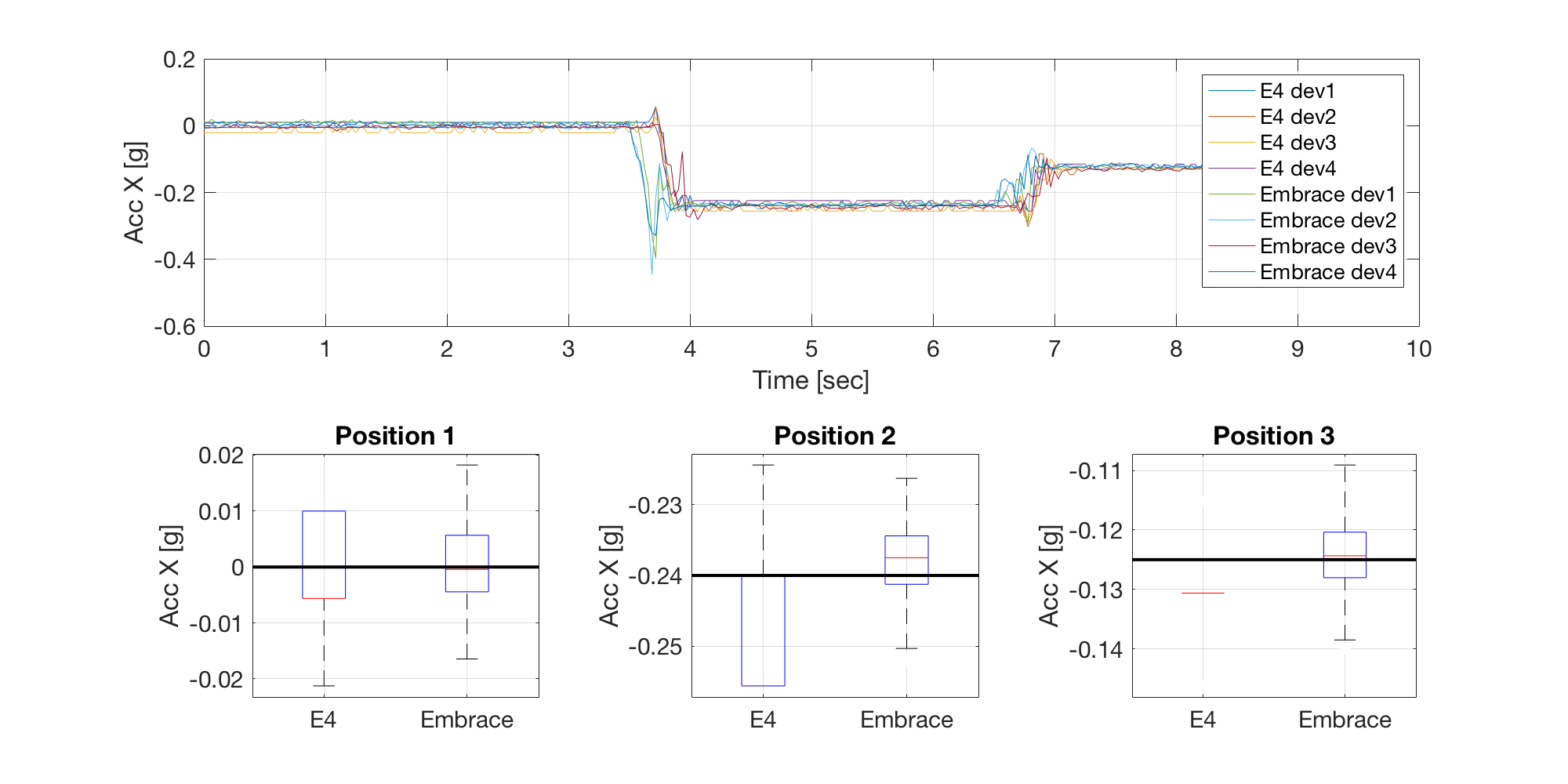
Figure 2. Top panel:** Raw data of the X axis from 4 Embrace and 4 E4 devices in 3 static positions. **Bottom-Right panel:** Box plot of X values measured by E4 and Embrace in the first static position. **Bottom-Center panel:** Box plot of X values measured by E4 and Embrace in the second static position. **Bottom-Left panel:** Box plot of X values measured by E4 and Embrace in the third static position. In all the box plots, the black line represents the reference value.

**
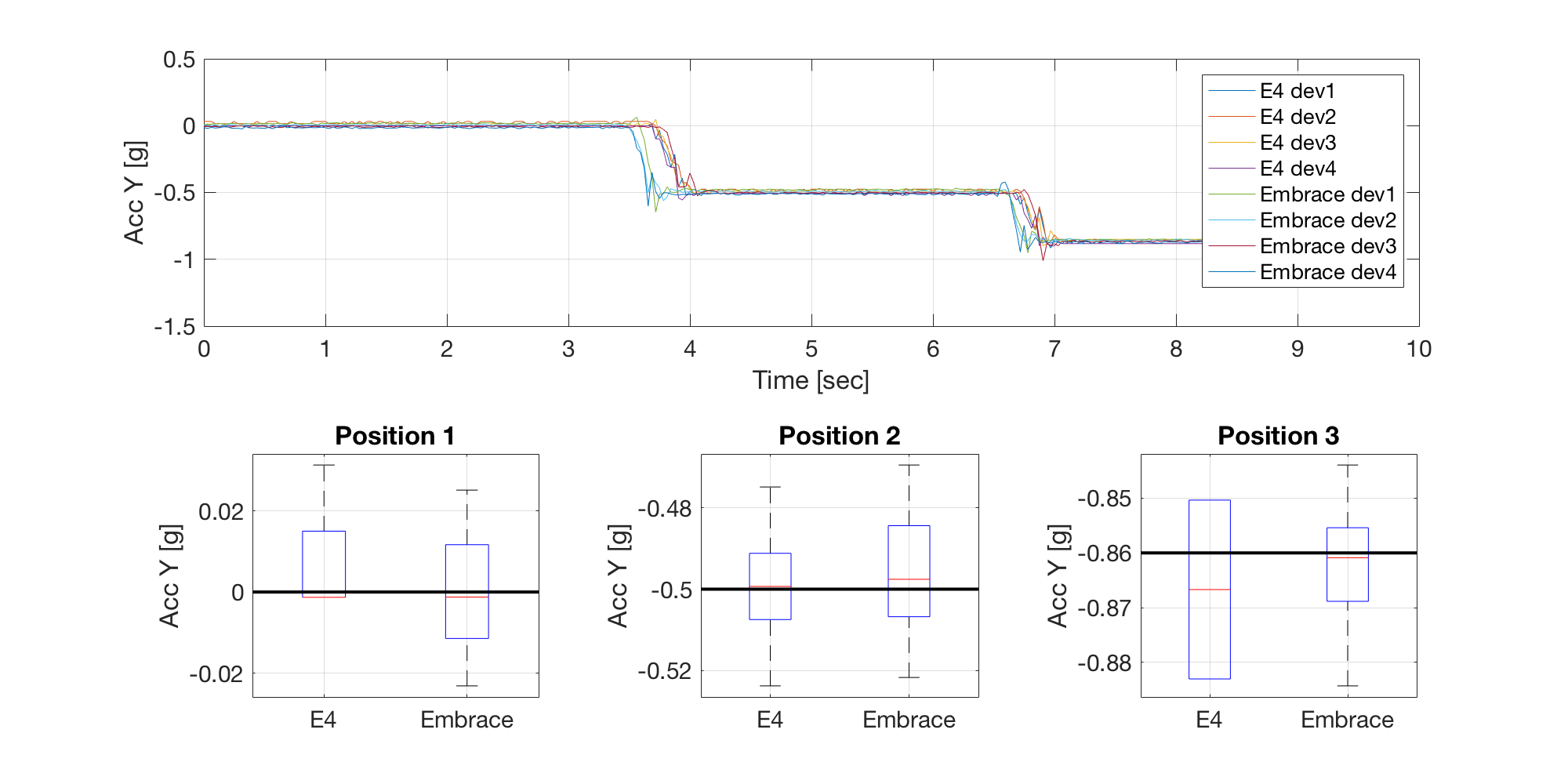
Figure 3. Top panel:** Raw data of the Y axis from 4 Embrace and 4 E4 devices in 3 static positions. **Bottom-Right panel:** Box plot of Y values measured by E4 and Embrace in the first static position. **Bottom-Center panel:** Box plot of Y values measured by E4 and Embrace in the second static position. **Bottom-Left panel:** Box plot of Y values measured by E4 and Embrace in the third static position. In all the box plots, the black line represents the reference value.

**
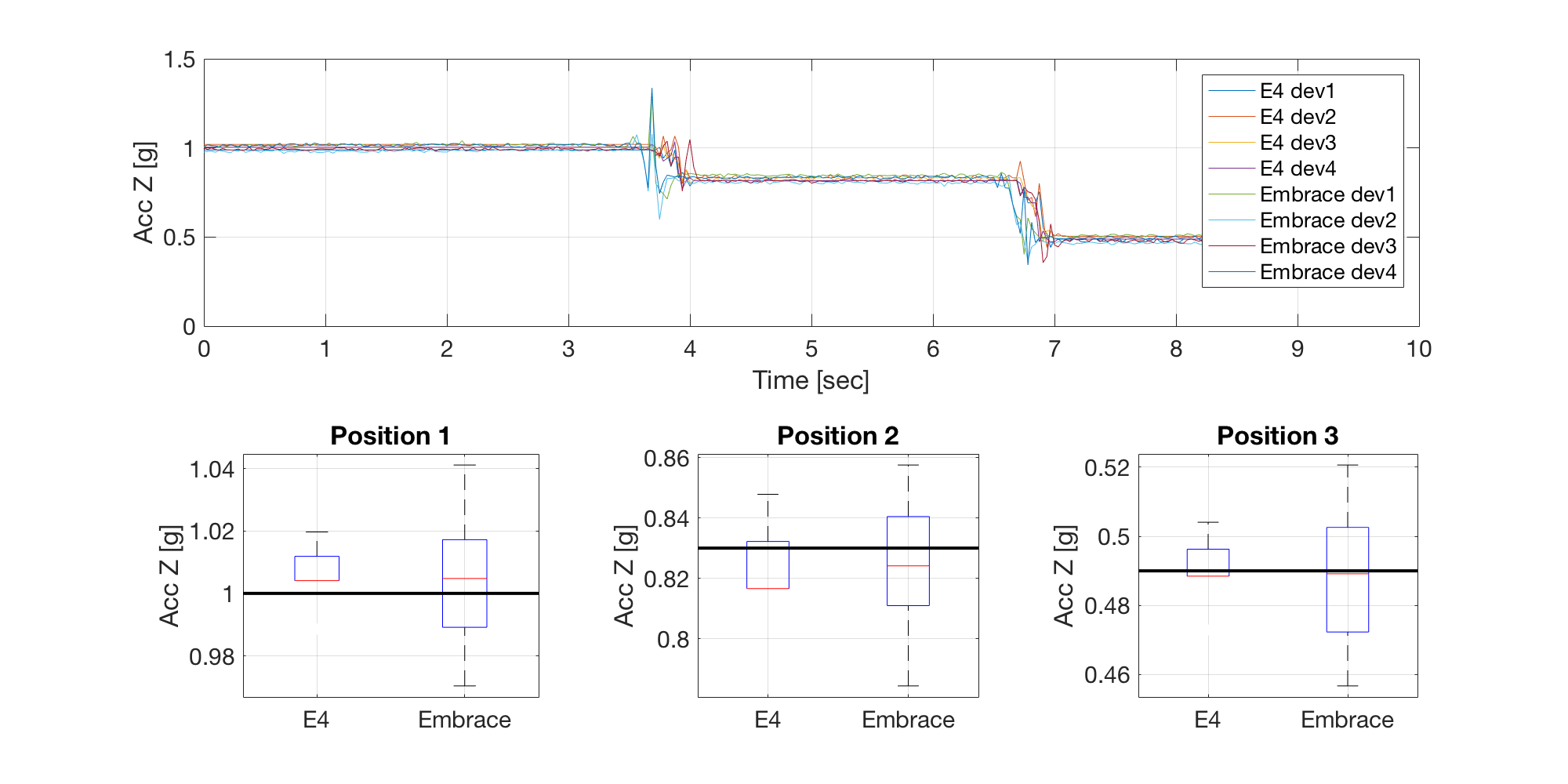
**

**Figure 4. Top panel:** Raw data of the Z axis from 4 Embrace and 4 E4 devices in 3 static positions. **Bottom-Right panel:** Box plot of Z values measured by E4 and Embrace in the first static position. **Bottom-Center panel:** Box plot of Z values measured by E4 and Embrace in the second static position. **Bottom-Left panel:** Box plot of Z values measured by E4 and Embrace in the third static position. In all the box plots, the black line represents the reference value.

Table 2 summarizes the absolute error (g), precision (g) and accuracy (%) of ACC measurements of the two devices, with respect to the known acceleration vector at the 3 static positions.

|  | **Absolute error E4 (g)** | **Absolute error Embrace (g)** | **Precision E4 (g)** | **Precision Embrace (g)** | **Accuracy E4 (%)** | **Accuracy Embrace (%)** |
| --- | --- | --- | --- | --- | --- | --- |
| **X axis** | 0.006 | 0.004 | 0.005 | 0.005 | 2.12 | 2.18 |
| **Y axis** | 0.009 | 0.009 | 0.008 | 0.010 | 1.49 | 2.28 |
| **Z axis** | 0.011 | 0.014 | 0.011 | 0.011 | 1.15 | 2.28 |

**Table 2.** The first four columns represent the absolute error (g), expressed as median, and the precision (g), expressed as interquartile range, of the E4 and Embrace in measuring the gravity component in the 3 static positions for each ACC axis respectively. The last two columns show the accuracy, the median of the absolute error between the measured values from the 3 axes for both E4 and Embrace and the real value, expressed as a percentage with respect to the real value.

**2.2.2 Dynamic tests**

Figure 5 shows the comparison of the raw data from the 8 devices considered (left column) for each sensor axis (rows). Overall, the measured temporal profiles of acceleration match well (i.e., they are overlapped), especially along the Y and Z axes, which are the most influenced by the movement. The X axis is subjected to a lower acceleration; therefore, the observed excursion is inferior and the recordings are noisier. The spectral analysis (right column) shows that the informative content is preserved and constant across the different tested devices.

The maximum absolute error in the computation of the main frequency (with respect to the known frequency applied with the test jig) is equal to 10.5 mHz (Table 3).


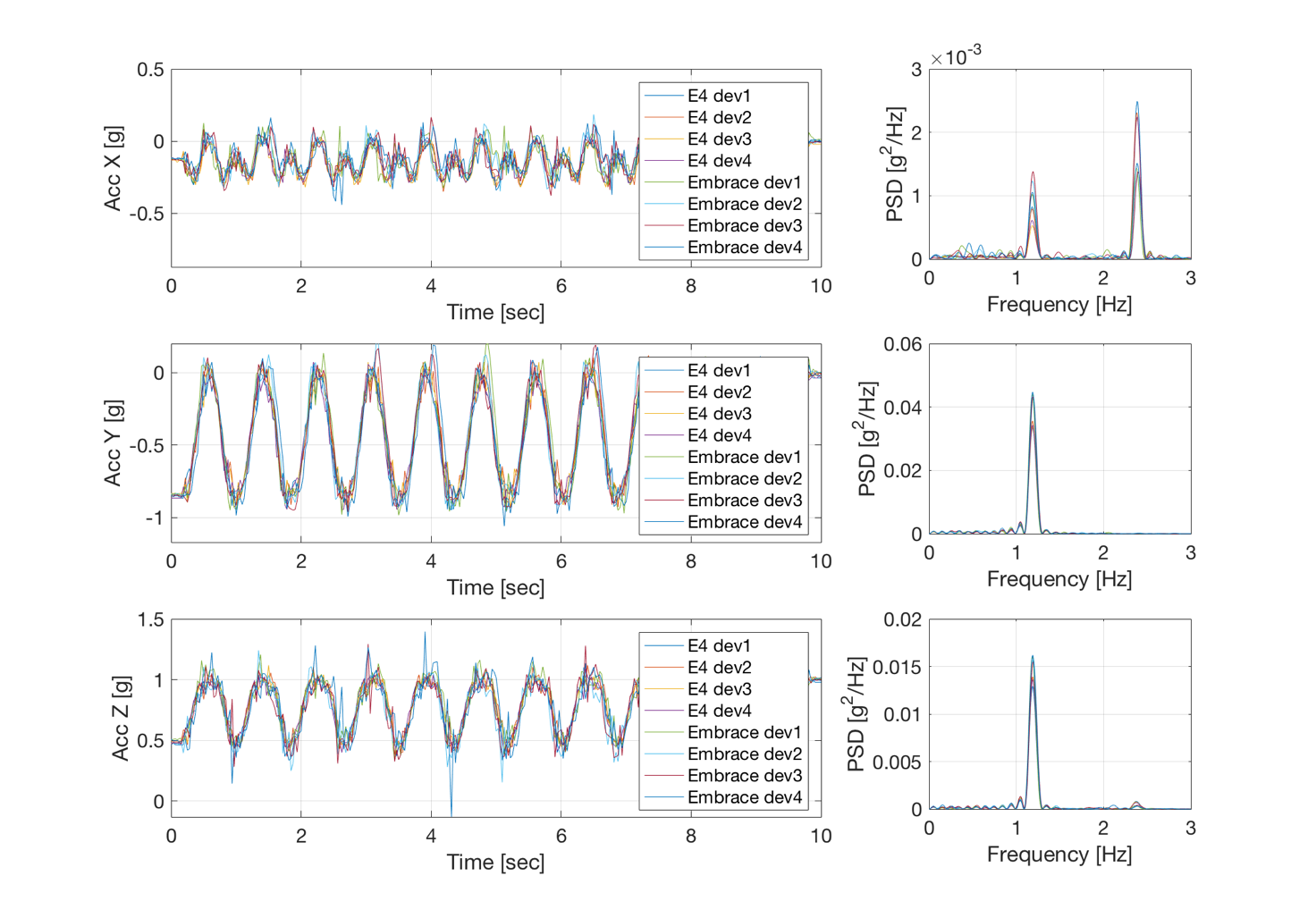
**Figure 5. Left Column:** Raw data of the 3D accelerometers of 8 devices (X, Y and Z axes represented in top, center and bottom graph, respectively). **Right Column:** Spectral Analysis of the ACC data (X, Y and Z axes represented in top, center and bottom graph, respectively).

|  |  | **Min Difference [Hz]** | **Mean Difference [Hz]** | **Max Difference [Hz]** |
| --- | --- | --- | --- | --- |
| **X axis** | **E4** | 0.0105 | 0.0105 | 0.0105 |
|  | **Embrace** | 0.0027 | 0.0046 | 0.0105 |
| **Y axis** | **E4** | 0.0027 | 0.0027 | 0.0027 |
|  | **Embrace** | 0.0027 | 0.0052 | 0.0105 |
| **Z axis** | **E4** | 0.0027 | 0.0027 | 0.0027 |
|  | **Embrace** | 0.0027 | 0.0059 | 0.0105 |
| **TOTAL** | | 0.0027 | 0.0053 | 0.0105 |

**Table 3.** Minimum, average and maximum absolute error (Hz) in computing the main frequency for every accelerometer axis and device.

The Bland-Altman analysis (Figure 6) shows a high correlation between the two sensors (r^2^=0.9674, left graph) with a bias that is close to zero (bias=0.0088 g, right graph).


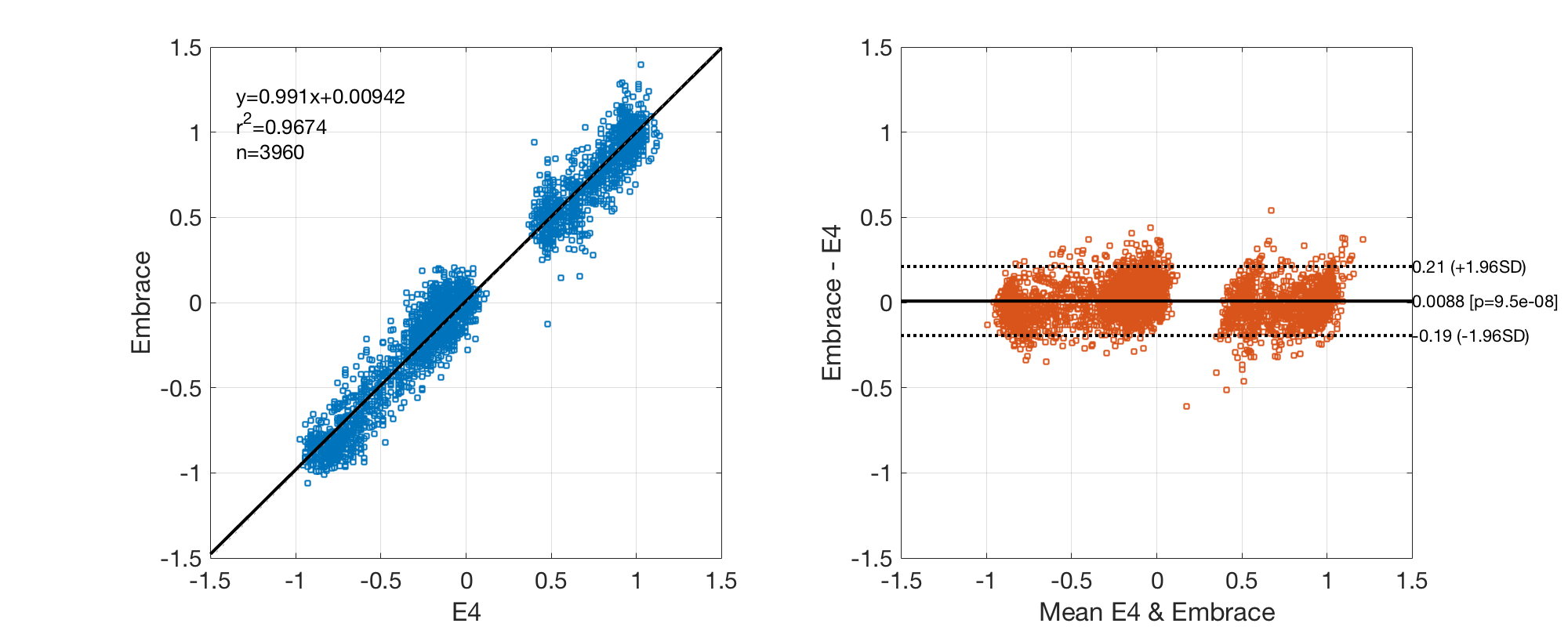


**Figure 6. Left Graph:** Correlation analysis between E4 and Embrace. Data points are acceleration samples pooled among the 4 pairs of devices tested and among the 3 axes. **Right Graph:** Bias analysis between E4 and Embrace data.

**2.3 Conclusions**

Data acquired with Embrace and E4 3-axis accelerometers showed a comparable accuracy (errors less than 3%) during static tests. Both absolute errors and precision achieved the desired low values. Regarding the dynamic test, the estimation of the main frequency using a simple periodogram approach showed a maximum error of 10.5 mHz on both the devices for each accelerometer axis, validating the use of both devices for achieving similar spectral components extraction.

1. **References**

Boucsein W. Electrodermal Activity. Boston, MA: Springer US; 2012

ISO 5725-1, 1994. Accuracy (Trueness and Precision) of Measurement Methods and Results e Part 1: General Principles and Definitions.

Picard RW, Fedor S, Ayzenberg Y. Multiple arousal theory and daily-life electrodermal activity asymmetry. Emotion Review. 2016;8(1):62–75.

Poh M-Z, Loddenkemper T, Reinsberger C, Swenson NC, Goyal S, Madsen JR, et al. Autonomic changes with seizures correlate with postictal EEG suppression. Neurology. 2012 Jun;78(23):1868–76.

Sarkis RA, Thome-Souza S, Poh M-Z, Llewellyn N, Klehm J, Madsen JR, et al. Autonomic changes following generalized tonic clonic seizures: An analysis of adult and pediatric patients with epilepsy. Epilepsy Res. 2015 Sep;115:113–8.
